# Determinants of Successful Implementation of Assistive Technologies for Dementia: Exploratory Survey

**DOI:** 10.2196/53640

**Published:** 2024-09-13

**Authors:** Henriëtte Geralde Van der Roest, Hannah Liane Christie, Manuel Angel Franco-Martin, Rose-Marie Dröes, Marjolein Elizabeth de Vugt, Franka Meiland

**Affiliations:** 1 Department on Aging, Netherlands Institute of Mental Health and Addiction (Trimbos Institute), Utrecht, Netherlands; 2 Alzheimer Center Limburg, Department of Psychiatry and Neuropsychology, Mental Health and Neuroscience Research Institute, Faculty of Health, Medicine and Life Sciences, Maastricht University, Maastricht, Netherlands; 3 Department of Psychiatry and Mental Health, Salamanca University, Zamora, Spain; 4 Department of Psychiatry, Amsterdam UMC (Vrije Universiteit Amsterdam location), Amsterdam Public Health Research Institute, Amsterdam, Netherlands; 5 Department of Medicine for Older People, Amsterdam UMC (Vrije Universiteit Amsterdam location), Amsterdam Public Health Research Institute, Amsterdam, Netherlands

**Keywords:** assistive technology, dementia, implementation, caregiving, psychosocial research

## Abstract

**Background:**

Despite positive results for the use of assistive technologies (ATs) in dementia, the uptake of ATs lags behind. It is considered important to assess determinants of successful or unsuccessful implementation of ATs.

**Objective:**

We explored factors that influence the implementation of ATs for community-dwelling people with dementia, with the aim to better understand potentially effective implementation strategies.

**Methods:**

A cross-sectional survey for researchers was developed and disseminated, exploring factors that influence either successful or unsuccessful implementation of ATs for dementia. The survey consisted of closed and open questions.

**Results:**

The response rate was 10% (21/206); the 21 respondents who completed the survey were from 8 countries. Determinants of implementation were described for 21 ATs, of which 12 were successfully and 9 were unsuccessfully implemented. Various types of ATs were included, such as online platforms, sensors, or physical aids. The main determinants of implementation success were related to the AT itself, contextual factors, research activities, and implementation strategies. There was a lack of research data on some ethical issues and cost-effectiveness.

**Conclusions:**

This study provided insight into some main barriers to and facilitators of implementation of ATs in dementia related to the AT itself, context, research-related activities, and applied implementation strategies. Lessons were formulated for various stakeholders to improve the implementation effectiveness of ATs in dementia.

## Introduction

The importance of assistive technology (AT) in the delivery of health care and support is increasingly being recognized [1,2]. AT in dementia care has been defined as “any item, piece of equipment, product or system driven by electronics, whether acquired commercially, off-the-shelf, modified or customized, that is used to help persons with dementia in dealing with the consequences of dementia” [3,4]. A perceived advantage of AT is improved access to care, even for people in remote areas. AT can make it easier to provide customized care, can be more entertaining, and can save time and costs [5-10]. Specifically for people with dementia and their carers, positive effects have been reported from the use of AT. For people with dementia, AT has been found to positively affect, for example, cognition, task performance, participation in enjoyable and meaningful activities, social contacts, self-esteem and well-being, behavioral and psychological symptoms, and safety [4,11-13]. For family carers, the following benefits have been reported: feelings of reassurance, lessened burden, improved self-efficacy, more caregiving knowledge, and improved mental health [11,14,15]. Furthermore, systematic literature reviews on technology-based dementia education for professional carers showed positive effects on dementia knowledge, readiness to change, receptiveness to training, communication skills, and self-efficacy [13,16]. Finally, the potential of AT to improve access to dementia care is even more crucial given the context of the COVID-19 pandemic. During this period, AT provided an alternative means of support for closed day programs and services, as well as an alternative to the downscaled professional and social support for people with dementia and their caregivers. Recent articles have advised care professionals to use technology to organize support remotely [13,17].

Despite these positive results, the uptake of AT in dementia care has been disappointing. Various issues may account for this, such as relevant stakeholders and potential users not being included early on in the development of ATs, lack of high-quality effectiveness and cost-effectiveness studies, lack of easily accessible information about existing ATs, and negative attitudes toward technology among family and professional caregivers [4,18-20]. Numerous implementation models and theories have been developed to help identify and map factors that influence implementation, such as the nonadoption, abandonment, scale-up, spread, sustainability framework [21] and the Consolidated Framework for Implementation Research [22]. These factors include the characteristics of the innovation (eg, added value as compared to regular support, the development process, and reliability and ease of use), characteristics of the adopters (professionals) and the end users (people with dementia and family carers), characteristics of the context (organization, social setting, community, laws, and regulations), characteristics of the implementation process (implementation strategies used), and implementation outcomes. In a market study in the European Union, it was concluded that there are 7 potential categories of barriers to the uptake of AT: cultural, regulatory and policy, social security, industrial and technical, knowledge, financial, and market related. These types of barriers exist in all European countries but differ across countries in the extent to which they affect uptake [2]. This underscores the need to tailor AT implementation to specific contexts.

In this paper, we report on a study in which we explored factors that influence the implementation of AT for community-dwelling people with dementia, with the aim to better understand potentially effective implementation strategies. Implementation was defined as “the process of putting to use or integrating evidence-based interventions within a setting” [23]. We targeted community-based dementia care settings and focused on ATs that aim to promote social health in people with dementia, that is, technologies that intend to (1) help people living with dementia manage their everyday lives across the disease journey, such as electronic calendars and reminders for activities, medication reminders, aids to perform activities of daily life, robots, and navigation systems; (2) help people to engage in meaningful and pleasurable activities, such as cognitive stimulation and physical activities; and (3) improve participation in social and meaningful activities [24].

The objective of this exploratory study was to describe factors that determine (1) successful implementation of evidence-based ATs and (2) unsuccessful implementation of ATs. Based on understanding the determinants of successful or unsuccessful implementation, we made a set of recommendations for the benefit of various stakeholders, including researchers who wish to bring their ATs into practice, clinicians who wish to be informed about, and implement, best practices in AT, and policy makers who need to make decisions about effective ways to fund development, research, and implementation of ATs.

## Methods

### Design

A cross-sectional design was used in which data were collected digitally through a web-based survey accessible from October until December 2019. On the survey platform, all participants received an information letter explaining the aims of the study and guaranteeing the pseudonymous processing of their data and responses, as well as the option to discontinue participation in the study at any time. To ensure the anonymity of the participants, the studied interventions are not reported by name; only their characteristics are described.

The survey was sent out in October 2019 via email, and subsequently 2 reminders were sent after 2 and 4 weeks. Participants could decide for themselves whether they completed the survey on successful implementation of AT, unsuccessful implementation of AT, or both surveys.

### Participants and Setting

Participants were recruited via the European Interdem network, which consists of psychosocial researchers from 19 countries in Europe and beyond, working together in the field of early, timely, and quality psychosocial interventions in dementia [25], as the objective of the survey was to map the determinants of the implementation of AT interventions for people with dementia that were developed in a research context. As such, researchers from this network are best placed to provide an overview of such interventions. The survey was sent out to all Interdem members (n=206). Those who were or have been working on projects on AT aiming to support people with dementia in the community were invited to participate. Members were also invited to forward the survey to colleagues known to work in this area (snowball sampling).

### The Survey

The survey was developed based on the literature on implementation and on expert opinions. From the literature, we proposed 5 categories of influencing factors: characteristics of the innovation, the adopters and end users, contextual factors, implementation strategies, and outcomes of the implementation [26-28]. In addition, expert opinions about the content of the survey were collected during a symposium organized by the Interdem Taskforce Assistive Technologies at the Alzheimer Europe conference in 2018. The team of authors further finalized the survey with this input. Also, background characteristics of respondents were included, such as country of residence, type of work organization and area of work, job description, gender, and age. As this was a new survey designed for the purposes of this study, it has not been psychometrically validated as of yet.

The surveys for both successfully and unsuccessfully implemented ATs contained 67 identical structured and open-ended items regarding factors that potentially influence the implementation of ATs as described by respondents (Table 1).

Both surveys were programmed in dedicated survey software (QDNA version 1.1.4; ZorgDNA BV).

**Table 1.** Survey themes and subthemes.

Themes	Subthemes
Characteristics of the AT^a^	The aim of the AT with respect to the 3 areas of promoting social health Type of device Dementia-specific development Pricing Training requirements User-friendliness (personalization options, required support)
Characteristics of adopters and end users	End user of the AT Ownership of the AT Main beneficiary Required training or user criteria
Contextual factors	Care setting Policies and legislation Ethical issues
Implementation strategies	Stakeholder involvement Conducted studies on usefulness, effectiveness, cost-effectiveness, and implementation Presence of business plan; financing
Outcomes of the implementation	Scale of uptake of the AT; facilitators and barriers for implementation

a AT: assistive technology.

### Analyses

Descriptive statistics were used to describe the results of the survey and to explore differences between successful and unsuccessful implementations. Due to the small sample size and relatively large number of parameters, statistical tests could not be executed. The qualitative responses to the survey’s open-ended items provided only additional information to the quantitative data, such as to clarify a type of device used in the AT, and these responses were not analyzed separately.

### Ethical Considerations

This study did not fall under the scope of the Medical Research Involving Human Subjects Act. Therefore, ethical approval by an institutional review board was not obtained. The respondents were informed of the purpose of the study before the survey and gave their consent to the survey data being collected and analyzed pseudonymously (it was not possible to guarantee full anonymity during analyses because the AT was discussed by name by study participants—for many ATs, it is possible to ascertain which researchers or technological developers were behind their development). All AT names were removed from the final data, allowing for pseudonymous reporting of the results. The respondents did not receive any compensation.

## Results

### Respondents

Thirty-two of the 206 invitees declined to participate as they were not active in the field of AT; the survey was forwarded to at least 13 other researchers. The response rate was 10%; 21 of 206 respondents completed the web-based survey. The participants were employed in a variety of settings: 17 respondents indicated that they were employed in a university setting, 4 in a hospital setting, 4 in care organizations, and 1 in a research organization (nonacademic). Fifteen of the respondents described their main area of work as research, 5 as clinical (1 with inpatients and 4 with outpatients), and 1 as policy. The mean age of the respondents was 49 (SD 12) years, and 71% (15/21) were female. Respondents were from the Netherlands (n=9), the United Kingdom (n=4), Germany (n=2), the Czech Republic (n=1), Denmark (n=1), France (n=1), Hong Kong (n=1), and Italy (n=1). One response was missing.

### Included ATs

All 21 respondents filled in the survey for a single AT. However, 2 respondents independently filled in the survey for the same AT, so the total of number of ATs included in this study was 20. Table 2 is an overview of the types of ATs included.

The respondents described the implementation of 12 of these ATs as successful, while 9 were described as unsuccessful. Successfully implemented ATs were associated with national uptake (7/12, 58%), as opposed to international (1/12, 8%) or regional (3/12, 25%) uptake. Only 2 of the 9 unsuccessfully implemented ATs were deployed on a national level (22%).

**Table 2.** Types of assistive technology (AT) included.

Type of AT	Responses (n=21), n	
	Successful (n=12)	Unsuccessful (n=9)
Online platform	6	4
Robot	0	1
Monitoring, sensors, or GPS	3	1
Physical aids	2	2
Virtual reality	1	1

### Characteristics of the ATs

The survey responses showed that the majority of ATs were aimed at the management of everyday life (15/21, 71%). ATs that were available through multiple devices were more often implemented successfully. The devices most often used in successfully implemented ATs were personal computers (9/12, 75%), smartphones (7/12, 58%), and tablets (5/12, 42%). The range of devices used in unsuccessfully implemented ATs was less diverse, with tablets being reported most frequently (5/9, 56%). Moreover, mobile devices were more often implemented unsuccessfully, while apps were more often implemented successfully. The AT was not specifically developed for people with dementia in all cases; one-third to a quarter were described as “mainstream technology.”

Successfully implemented ATs tended to be more often developed specifically for people with dementia. An association between implementation success and personalization options of the AT was not clear. The design of almost all successfully implemented ATs was considered good or very good; the design of unsuccessfully implemented ATs was considered good by 56% (5/9) of the respondents and bad or very bad by 33% (3/9; the remainder were missing).

Successfully implemented ATs were more often freely available or considered as value for money and deemed affordable for everybody, while unsuccessfully implemented ATs were considered expensive or very expensive by one-third of the respondents. In contrast to the majority of the successfully implemented ATs, none of the unsuccessfully implemented ATs were described as functioning stably. Table 3 provides an overview of how the various device characteristics related to AT implementation success.

**Table 3.** Characteristics of the assistive technologies (ATs) and their contributions to implementation success. Note: missing answers are not reported but are taken into account in the calculation of the overall percentages per category (successful ATs and unsuccessful ATs).

AT characteristics		Successfully implemented ATs (n=12),n (%)	Unsuccessfully implemented ATs (n=9),n (%)
**Aim** ^**a**^			
	Fulfillment of one’s potential	3 (25)	1 (11)
	Management of everyday life	9 (75)	6 (67)
	Participation in social activities	1 (8)	2 (22)
**Supporting device**			
	Multiple devices	10 (83)	5 (56)
	PC	9 (75)	3 (33)
	Television	2 (17)	0 (0)
	Tablet	5 (42)	5 (56)
	Smartphone	7 (58)	2 (22)
	Smartwatch	1 (8)	0 (0)
	Actuator	1 (8)	0 (0)
	Sensors	3 (25)	2 (22)
	Camera	2 (17)	0 (0)
	Other	3 (25)	3 (33)
**Type of AT** ^**a**^			
	Mobile	1 (8)	4 (44)
	Stand-alone	2 (17)	2 (22)
	Integrated	2 (17)	3 (33)
	Software/app	6 (50)	3 (33)
**Specificity of the AT**			
	Specifically for people with dementia	9 (75)	5 (56)
	Mainstream	3 (25)	3 (33)
**Personalization options**			
	Yes, to a large extent	5 (42)	2 (22)
	Yes, to some extent	3 (25)	3 (33)
	No	4 (33)	3 (33)
**Design**			
	Very good	3 (25)	0 (0)
	Good	8 (67)	5 (56)
	Bad or very bad	1 (8)	3 (33)
**Pricing**			
	Freely available	6 (50)	3 (33)
	Value for money	4 (33)	0 (0)
	Expensive or very expensive	0 (0)	3 (33)
**Affordability**			
	For everybody	8 (67)	2 (22)
**Stability**			
	No problems	9 (75)	0 (0)

a Multiple answers could be provided.

### Characteristics of the Adopters and End Users

Approximately half of all ATs were owned by the person with dementia or informal caregiver. This was not clearly associated with implementation success. However, successfully implemented ATs were slightly more often owned by a care organization. Successfully implemented ATs were more often deemed affordable to everybody than unsuccessfully implemented ATs.

ATs mainly benefiting the person with dementia were more often successfully implemented. If criteria were defined for users, this was associated with successful implementation of ATs. Relatively more unsuccessfully implemented ATs required a preassessment to advise on or tailor the AT to the user. Most of the ATs required training to be used well. Unsuccessfully implemented ATs slightly more often required training of informal and professional carers. Few ATs could be managed independently by the person with dementia, and support by an informal carer was more often associated with successfully implemented ATs (Table 4).

**Table 4.** Characteristics of adopters and end users. Note: missing answers are not reported but are taken into account in the calculation of the overall percentages per category (successful assistive technologies [ATs] and unsuccessful ATs).

Characteristics of adopters and end users		Successfully implemented ATs (n=12),n (%)	Unsuccessfully implemented ATs (n=9),n (%)
**Ownership** ^**a**^			
	Person with dementia	6 (50)	5 (56)
	Informal carer	6 (50)	3 (33)
	Care organization	4 (33)	1 (11)
	Hospital	1 (8)	0 (0)
**Main beneficiary**			
	Person with dementia	7 (58)	4 (44)
**User criteria**			
	Defined	10 (83)	6 (67)
**Preassessment**			
	Needed	3 (25)	4 (44)
**Required training** ^**a**^			
	None	3 (25)	3 (33)
	Person with dementia	6 (50)	3 (33)
	Informal carer	6 (50)	5 (56)
	Professional carer	5 (42)	5 (56)
**Required support for person with dementia** ^**a**^			
	None	2 (17)	3 (33)
	From informal carer	9 (75)	4 (44)
	From professional carer	4 (33)	2 (22)

a Multiple answers could be provided.

### Contextual Factors

Most of the ATs were used in the home environment, without clear differences between successfully and unsuccessfully implemented ATs. Furthermore, interventions that were described as nonintrusive home ATs were more often seen as successfully implemented. Stable, continuous Wi-Fi was associated with successful implementation. Finally, while the majority of ATs were used within the home context, this was not associated with successful implementation. In contrast, ATs used outdoors were more often associated with successful implementation. When alternative products were available that addressed similar needs as the new AT, the AT was slightly more associated with successful implementation. Successfully implemented ATs were more often reimbursed by insurance or benefits or were paid by a care organization (Table 5).

**Table 5.** Contextual factors. Note: missing answers are not reported but are taken into account in the calculation of the overall percentages per category (successful assistive technologies [ATs] and unsuccessful ATs).

Contextual factors		Successfully implemented ATs (n=12),n (%)	Unsuccessfully implemented ATs (n=9),n (%)
**User setting** ^**a**^			
	Home	9 (75)	8 (89)
	Outside	4 (33)	1 (11)
	Day center	1 (8)	0 (0)
**Intrusiveness**			
	Nonintrusive	11 (92)	6 (75)
**Connection**			
	Continuous internet/Wi-Fi needed	8 (67)	3 (33)
**Alternative AT**			
	Available	7 (64)	4 (44)
**Means of financing** ^**a**^			
	Out of pocket	2 (17)	3 (33)
	Public health care insurance	4 (33)	1 (11)
	Social welfare benefits	1 (8)	1 (11)
	Care organization	3 (25)	0 (0)
	For rent	1 (8)	1 (11)
	For free	3 (25)	2 (22)

a Multiple answers could be provided.

### Implementation Strategies

#### Stakeholder Involvement

The findings on the ATs’ stakeholder involvement indicate that, in general, the involvement of people with dementia, informal caregivers, commercial parties, researchers, and care organizations was associated with successful implementation. Only the involvement of governments did not seem to affect implementation success (Table 6).

**Table 6.** Stakeholder involvement and assistive technology (AT) implementation success.

Stakeholder group involvement	Successfully implemented ATs (n=12), n (%)	Unsuccessfully implemented ATs (n=9), n (%)
Person with dementia	6 (50)	2 (22)
Informal caregiver	7 (58)	2 (22)
Commercial party	7 (58)	4 (44)
Government	3 (25)	2 (22)
Research	9 (75)	5 (55)
Care organization	7 (58)	2 (22)

#### Research Activities

It appears that ATs whose user friendliness has been studied are more often successfully implemented (10/12, 83% vs 4/9, 44%), independent of the outcome of the study. Also, effectiveness studies were more often part of the development process in successfully implemented ATs (7/12, 58%) than in unsuccessful trajectories (2/9, 22%). Few cost-effectiveness studies were performed—for just 4 ATs in total—and this was not associated with implementation success. Ethical issues were considered for 57% (12/21) of all included ATs. This happened equally often for successfully and unsuccessfully implemented ATs. Most respondents referred to the obtainment of medical ethical approval; for one study, AT ethical issues were registered, while another study addressed potential ethical issues in an intervention protocol. Ethics were considered most often in the development phase of ATs and least often during the implementation phase, in 25% (3/12) and 11% (1/9), respectively, of successfully and unsuccessfully implemented ATs. Regarding privacy and data security, these were taken into account for most ATs (16/21, 76% in total) and were not associated with implementation success. However, studies on barriers to and facilitators of implementation and the presence of a business plan were associated with successfully implemented ATs (6/12, 50% and 5/12, 42% for successfully implemented ATs, respectively, vs 2/9, 22% and 1/9, 11% for unsuccessfully implemented ATs).

### Main Facilitators and Barriers

The respondents were also asked to select a maximum of 5 facilitators considered as most important and a maximum of 5 most relevant barriers out of 30 factors that applied to the implementation of their ATs.

Facilitators that were most often mentioned were associated with the AT itself, including user-friendliness, the type of device (ie, personal computers, smartphones, and tablets were more often successfully implemented), and the availability of the AT on multiple devices. For successfully implemented ATs, personalization options were also considered to facilitate implementation. Contextual factors were only considered relevant for successful implementation; availability of a continuous internet or Wi-Fi connection in particular appeared to be the major facilitator.

In general, barriers were mentioned more often in unsuccessfully implemented ATs. The main barriers were, foremost, the opposite of the facilitators: a lack of user-friendliness and the unavailability of the AT on multiple devices. Next, contextual factors, such as an internet connection, available alternatives, ethics, privacy and data security, intrusiveness of the AT, and policy and laws, were mentioned as factors impeding the implementation. No main barrier could be identified.

Also, for successfully implemented ATs, respondents reported that the lack of financing and business plans for the AT was a major barrier that needed to be addressed in the implementation strategy. On the other hand, barriers for unsuccessfully implemented ATs were related to not being effective or cost-effective and lacking a marketing strategy and business plan (Table 7).

**Table 7.** Frequency of mentioned main facilitators of and barriers to implementation of assistive technologies (ATs) per category.

	Facilitators		Barriers	
	Successfully implemented ATs (n=12), n (%)	Unsuccessfully implemented ATs (n=9), n (%)	Successfully implemented ATs (n=12), n (%)	Unsuccessfully implemented ATs (n=9), n (%)
Adopters	3 (25)	2 (22)	1 (8)	3 (33)
Technology	7 (58)	5 (56)	3 (25)	7 (78)
Contextual	4 (33)	0 (0)	3 (25)	5 (56)
End user	2 (17)	2 (22)	1 (8)	4 (44)
Implementation strategy	2 (17)	2 (22)	3 (25)	3 (33)

## Discussion

### Main Findings

This study used a cross-sectional design to survey Interdem researchers with experience in AT projects for people with dementia on factors that influence the implementation of AT. This resulted in 21 survey responses, describing 12 successfully and 9 unsuccessfully implemented ATs. The main finding was that certain characteristics of the AT itself, such as the type of device used and the user-friendliness of the AT, seemed to be the most important for implementation success. Usability is one of the most relevant issues for successful implementation of technology [29]. The results also showed that contextual factors, although they can contribute to an AT’s successful implementation, were mostly considered as barriers. Furthermore, research activities carried out during the development process of an AT, such as studies on the user-friendliness and effectiveness of the AT, were more often related to successful implementation. The findings add value to the existing literature by helping fill the identified gap in knowledge [29,30] on implementation determinants in AT for dementia. This study on AT implementation has resulted in several important lessons for researchers, clinicians, and policy makers that can be considered as guidelines for successful implementation of AT in dementia.

### Lesson 1: Enhancing Value

The first lesson concerns how specific characteristics of the AT device are associated with implementation success. First, the ability to use the AT on multiple devices proved to be an important facilitator. This enables users to select a device matching their preferences and their capability to operate it. The possibility of multiple devices could also be related to another finding, namely the benefit of the availability of multiple delivery options for implementation success, such as the possibility of using a device not only indoors but also outdoors. The finding that an AT’s flexible delivery on multiple devices was an implementation determinant is consistent with previous research that identified a lack of interoperability as the biggest barrier to innovation, as it is often very hard to integrate new ATs into existing systems and devices [31]. Second, the finding that an AT’s user-friendliness played a considerable role in implementation success is in line with the outcomes of a previous systematic review on the implementation determinants of ATs for dementia [29]. However, as previously advocated by Bennett et al [32], it is important to find a balance between ease of use (for example, few or only simple actions required by the user) and proper diligence in terms of privacy and data security. This involves a thorough analysis of the implications of an AT for human rights laws, such as impacts on freedom of movement or privacy. Services such as surveillance technology should be refined to find the best balance between privacy and usefulness [33]. In sum, it is recommended to enhance an AT’s value through specific device characteristics, such as interoperability, user-friendliness, and an ethical approach to data security.

### Lesson 2: Optimizing Fit

The survey showed the importance of involving different stakeholders to facilitate implementation success. Previous research has emphasized the need to involve people with dementia throughout the development, evaluation, and implementation of ATs for dementia [34]. However, this study also makes clear that successful implementation depends on a wide involvement of actors in the dementia care network. Rigorously mapping stakeholder needs for a particular AT not only contributes to a more appropriate business model and sustainability, it also facilitates person-centered care [35] by taking into account the needs of all persons involved in the dementia care network. Potential barriers and facilitators such as workload and lack of resources in formal care, as well as easy access to and affordability of the AT for informal carers can be considered timely [36]. Moreover, the study findings indicate that the same feature can both hinder or facilitate implementation, depending on the context. This implies that there is no universal rule for implementing ATs successfully, and it remains necessary to adapt each AT to its implementation context, resulting in a better fit [36,37]. The findings of this study thus suggest that people with dementia as well as other stakeholders should be engaged in the cocreation of new technologies for dementia and that new ways and methodologies should be developed to promote this cooperation [38]. In sum, it is recommended to optimize the fit to the target group or groups by involving a wide range of stakeholders, in addition to people with dementia, at every stage of AT development and evaluation.

### Lesson 3: Ensuring Equity and Sustainability

A third lesson is that the affordability of ATs in dementia care is important for equal access to care and, in turn, for sustainable implementation. This has also been stressed by Moyle [39], who pointed out that ATs can be quite expensive, as personalized solutions are preferred. A recurring criticism of ATs such as eHealth has been that they can foster exclusion, as users must have a high level of digital and mental health skills to engage with some ATs, as well as sufficient resources to purchase the required devices [40]. Moreover, the COVID-19 pandemic has exacerbated health inequalities around the world [41]. Not only did the disease itself lead to worse health outcomes in vulnerable populations, the limitations on human contact disproportionately affected older people, accelerated cognitive decline in people with dementia, and increased social isolation of informal carers [42,43]. ATs could prevent understimulation and support social contact at a distance. Thus, successful implementation of ATs is important.

A first way AT developers can mitigate these inequities in AT implementation for dementia is by ensuring affordability through business modeling [44]. Previous research has emphasized the benefits of preliminary business models for new ATs. A study of AT applications in dementia not only helped to determine (affordable) costs and revenues, but also showed that establishing a business model facilitates decision-making by potential implementers, as there is an indication that the AT will be available beyond the study period and that efforts to apply the AT will not be wasted [45]. A second way is for AT developers to emphasize the importance of rigorous research regarding evaluation and implementation that takes the diverse characteristics of potential target users into account. This study confirmed that the execution of certain research activities, such as research into user-friendliness and effectiveness, was in itself a success factor in AT implementation. An intersectional research approach would strongly contribute to better implementation of ATs in dementia. Intersectionality is centered around “a variety of multi-level interacting social locations, forces, factors and power structures that shape and influence human life,” which goes beyond gender-specific and social-determinant frameworks [46]. An intersectional approach can help broaden the impact of ATs intended for dementia care to a more diverse group by taking a wider range of needs into account and not reducing individuals to one defining characteristic, such as age, gender, migration status, education, sexuality, or ethnicity. It can also reduce implementation barriers by avoiding an unpersonalized, one-size-fits-all approach [47]. In sum, it is recommended to ensure equity and sustainability in AT implementation through an intersectional approach and business modeling.

### General Recommendations

The findings from this study have produced a number of useful lessons for a variety of stakeholders, including researchers who wish to bring their ATs into practice, clinicians who want to provide the most suitable AT to their clients, and policy makers who wish to know the most effective ways to fund AT development, research, and implementation. In general, based on these findings, we recommend considering alternative study designs that are flexible and suit the fast-paced, changing, and innovative nature of technology, as well as the progressive disease trajectory of dementia, to complement more rigid and resource-intensive designs, such as randomized controlled trials. These alternative study designs can iteratively and responsively examine the user-friendliness, effectiveness, cost-effectiveness, and sustainability of the implementation of ATs, with varying relevant outcome measures depending on the studied stakeholder group.

These recommendations for improving successful implementation of ATs in dementia care are in line with previous implementation research findings [48-50]. For example, Ienca et al [51] proposed a proactive approach, taking into account four normative principles that may help successful implementation: (1) minimization of power imbalances in decision-making, which could be achieved through cooperation among different stakeholders, including end users; (2) compliance with biomedical ethics, such as beneficence, nonmaleficence, autonomy, and justice; (3) translation of research into practice; and (4) raising social awareness and sharing knowledge across society. They additionally complement the recent recommendations made in the *INDUCT/DISTINCT Best Practice Guidance on Human Interaction with Technology in Dementia*, including its recommendations around business modeling and optimizing AT fit to the implementation context [30].

### Strengths and Limitations

A first major strength of this study is the multidisciplinary range of respondents’ professional backgrounds. Although it is not clear how representative the Interdem Network is of researchers in the field of psychosocial dementia care in Europe, the multidisciplinarity of the network, ranging from physicians (psychiatrists, geriatric care physicians, and neurologists); clinical, social, and neuropsychologists; social scientists; paramedics; palliative care specialists; and movement scientists, allowed us to potentially involve a broad group of participants from diverse backgrounds in this survey. A second major strength of this study is the combined use of qualitative and quantitative research methods to paint a rich and detailed picture of the current field of implementation of ATs for dementia. This richness of data also sheds light on what happens after the end of the research phase, during which the interventions are well controlled and care professionals are motivated to participate. Hence, the added value of this research to the existing literature is its assessment of implementation results in real environments, in addition to quantitative data.

This study also had some important limitations, which need to be mentioned. First, the study had a cross-sectional design, which does not allow for any causal interpretations of the associations found between the studied implementation determinants. Furthermore, the survey questionnaire is in its first iteration and not psychometrically tested. Nonetheless, this study constitutes a first, important step toward identifying relevant determinants of successful implementation of ATs in dementia care for future study. Second, this study had a rather limited sample size and large variety of ATs and surveyed countries, making it difficult to generalize the findings. Also, we do not know the exact response rate as we do not know how many Interdem members had done research with AT at the time the survey was conducted. Also, the small sample size may have resulted in a biased sample. As a result, the analysis of this exploratory study was limited to a descriptive approach. Altogether, 20 different ATs were considered by the respondents, with specific issues addressed. Given the involvement of the respondents in the different ATs, despite the small sample size, valuable information has been obtained in this study. Third, the terminology used in the survey in this study regarding “free-to-use” interventions may have led to different interpretations, making it hard to draw definitive conclusions on this. It is possible that this was interpreted as “without costs for the end user,” even though there are still costs for other parties, such as the implementing organizations or health insurers. Finally, this study focused only on a limited number of high-income implementation contexts. Therefore, the study’s insights may not all apply to a wider variety of contexts, such as low- and middle-income countries.

### Directions for Future Research

This study highlights several important areas for future research. First, while a number of implementation determinants were associated with successful implementation, future research could attempt to infer which strategies are the most effective in facilitating AT implementation by differentially evaluating these strategies in experimental designs tailored to specific types of AT and implementation contexts. Second, it would be useful to explore AT implementation in different contexts, including low- and middle-income countries. Finally, this study should be followed up, and participants should be asked to provide more qualitative first-hand data on their implementation experiences. In doing so, it will be important to ask people with dementia, informal carers, and care professionals about why they want or do not want to use these interventions.

### Conclusions

This study provides insight into some main facilitators of and barriers to successful implementation of ATs in dementia care, such as factors related to the AT itself, contextual factors, if accompanying research activities were done into the AT, and applied implementation strategies. Based on this, lessons and recommendations were formulated to improve the implementation of AT in dementia care.

## Abbreviations

AT: assistive technology
